# Supplementary material for: Selection of Promising Novel Fragment Sized S. aureus SrtA Noncovalent Inhibitors Based on QSAR and Docking Modeling Studies
Source: Molecules. 2021 Dec 19;26(24):7677. doi: 10.3390/molecules26247677 (PMC8709105; doi:10.3390/molecules26247677)
Supplement: Supplementary file 1 [file molecules-26-07677-s001.zip › Supporting_info/shulga_kudryavtsev_SI_ed2_applied.pdf]

Supporting information for

Selection of promising novel fragment sized *S. Aureus* SrtA noncovalent inhibitors based on QSAR and docking modeling studies

Dmitry A. Shulga[1,*], Konstantin V. Kudryavtsev[2,3,*]

[1]Department of Chemistry, Lomonosov Moscow State University, Leninskie Gory 1/3, 119991, Moscow, Russian Federation;

[2]Laboratory of Molecular Pharmacology, Pirogov Russian National Research Medical University, Ostrovityanova Street 1, 117997, Moscow, Russian Federation,

[3]Laboratory of Chemical Synthesis and Catalysis, Moscow Institute of Physics and Technology, Institutskiy per. 9, 141701, Dolgoprudny, Moscow Region, Russian Federation

*Correspondence: dmitry.a.shulga@gmail.com; konstantin@kudryavtsev.ru; Tel.: +79037640655

Tables S1-S4 and S11 are provided as separate tab-separated-value (TSV) file amenable for both Excel and machine learning analysis.

Table S1-S4 and S9 titles.

Table S1. A set of active compounds used for QSAR classification model building.

Table S2. A set of inactive compounds used for QSAR classification model building.

Table S3. A set of active compounds used for QSAR regression model building. The IC50 activity values ("Act" column) are in nM.

Table S4. A set of inactive compounds used for QSAR regression model building. The IC50 activity values ("Act" column) are in nM.

Table S11. A set of prospective ligands with calculated descriptor values.

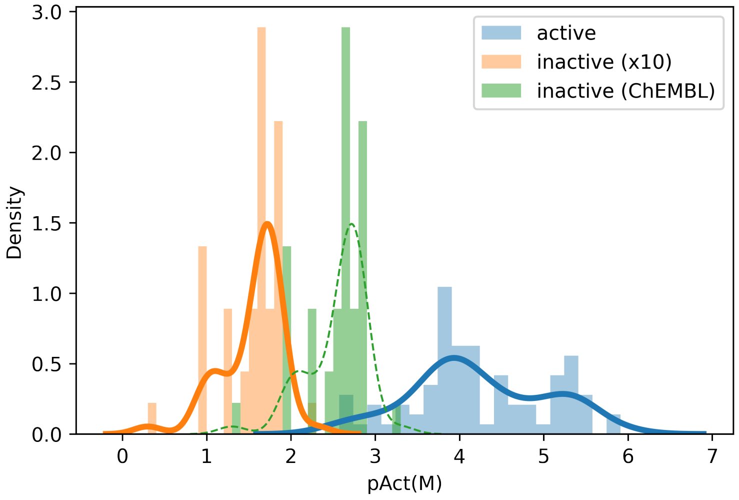

Figure S1. Distribution of activity of structures for regression model building. Activity is in -log10(Act, M). The array threshold values are provided in "inactive (ChEMBL)" set, whereas the adjusted data used in modeling are designated as "inactive (x10)".

Scan for the L1-regularization parameters

1. Classification models

1.1 Support vector machine (SVM)

The models were built using sklearn.svm.LinearSVC (https://scikit-learn.org/stable/modules/generated/sklearn.svm.LinearSVC.html), with penalty='l1' and dual=False settings. The regularization parameter C was varied. The strength of the regularization is inversely proportional to C.

Accuracy is the ratio of correctly predicted actives and inactives to the number of data points, accuracy = TP+TN/(TP+TN+FP+FN).

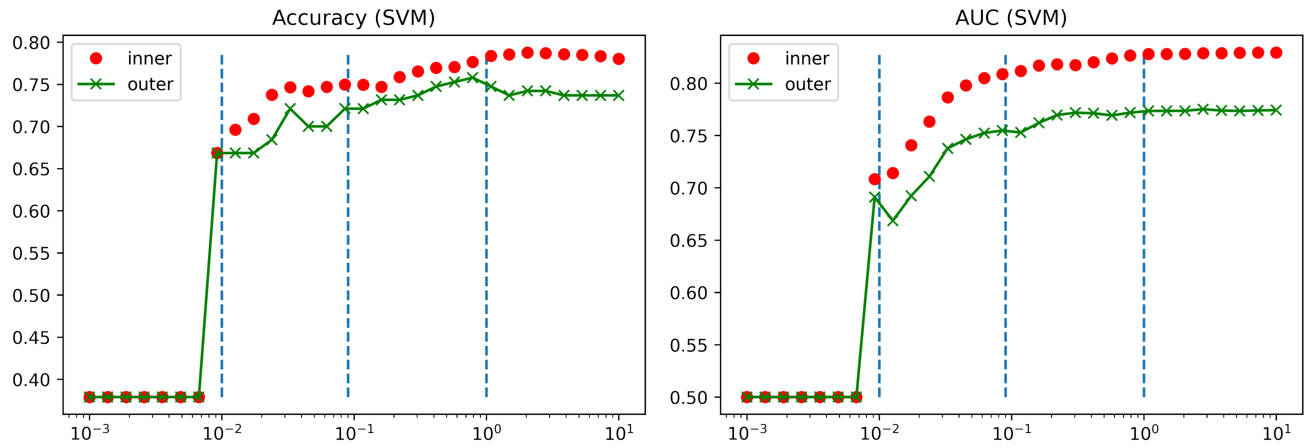

Figure S2. Dependence of the accuracy and the area under curve (AUC) on the regularization parameter C. The metrics are estimated on either the ‘inner’ (the training) set or the ‘outer’ (Leave-one-out) test. The vertical lines correspond to the three parameters used to produce three different models in the manuscript, corresponding to C=1e-2, 9e-2, 1.

1.2 Logistic regression (LR) classification

The model was built using sklearn.linear_model.LogisticRegression with penalty=”l1” and solver=”liblinear” (https://scikit-learn.org/stable/modules/generated/sklearn.linear_model.LogisticRegression.html). The regularization parameter C was varied. The strength of the regularization is inversely proportional to C.

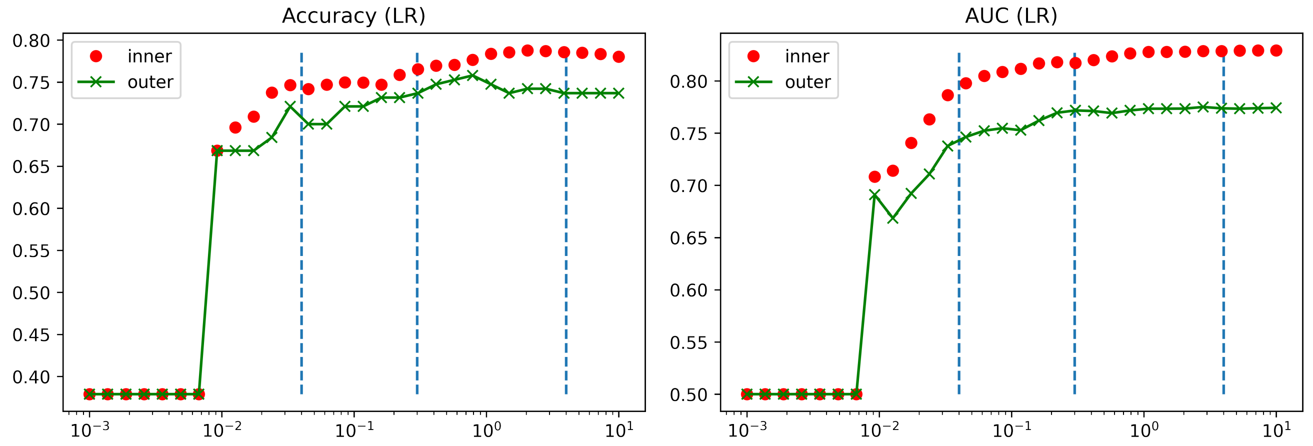

Figure S3. Dependence of the accuracy and the area under curve (AUC) on the regularization parameter C. The metrics are estimated on either the ‘inner’ (the training) set or the ‘outer’ (Leave-one-out) test. The vertical lines correspond to the three parameters used to produce three different models in the manuscript, corresponding to C=4e-2, 3e-1, 4.

2. Regression models

2.1 LASSO regression

The model was built using sklearn.linear_model.Lasso (https://scikit-learn.org/stable/modules/generated/sklearn.linear_model.Lasso.html). The regularization parameter alpha was varied. Larger values of alpha correspond to stronger regularization.

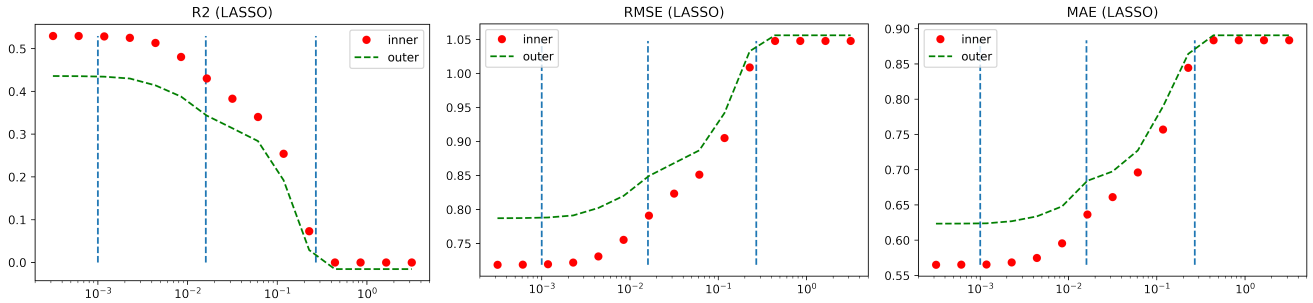

Figure S4. Dependence of R^2, RMSE and MAE on the value of regularization parameter alpha. The metrics are estimated on either the ‘inner’ (the training) set or the ‘outer’ (Leave-one-out) test. The vertical lines correspond to the three parameters used to produce three different models in the manuscript, corresponding to alpha=1e-3, 1.6e-2, 2.7e-1.

2.2 Support vector machine regression (SVR)

The model was built using sklearn.svm.SVR (https://scikit-learn.org/stable/modules/generated/sklearn.svm.SVR.html) using kernel="rbf". The regularization parameter C was varied (scaling L2-norm). The strength of the regularization is inversely proportional to C.

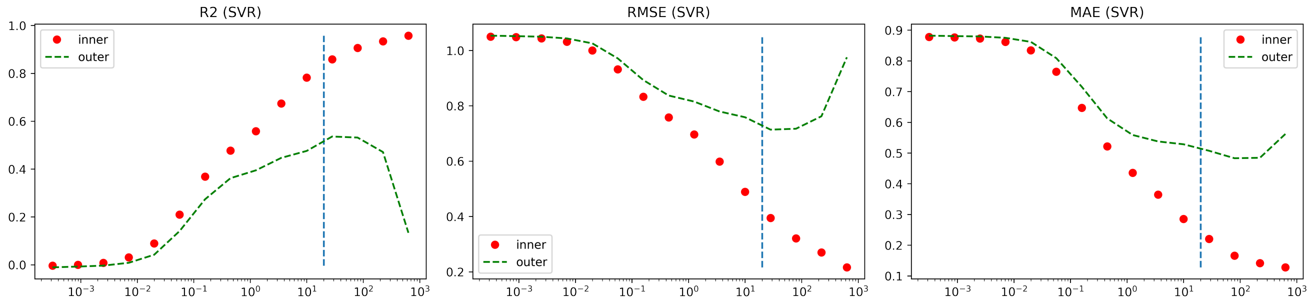

Figure S5. Dependence of R^2, RMSE and MAE on the value of regularization parameter alpha. The metrics are estimated on either the ‘inner’ (the training) set or the ‘outer’ (Leave-one-out) test. The vertical line correspond to the parameter value used to produce the model in the manuscript, corresponding to C=20.

Table S5. Correlation of the descriptors with activity for classification model molecule set and final decision on using descriptors in model building.

#	Descriptor	Mean value (std. dev.), unscaled	Correlation	Use in models
1	MolLogP	3.3 (1.8)	-0.36	yes
2	TPSA	71.7 (32.7)	0.10	no
3	NHOHCount	1.8 (1.5)	-0.093	no
4	NOCount	5.0 (2.0)	0.13	no
5	NumHAcceptors	4.3 (1.9)	0.18	yes
6	NumHDonors	1.8 (1.3)	-0.12	yes
7	NumRotatableBonds	5.4 (3.9)	-0.032	yes
8	NumHeteroatoms	5.7 (2.5)	0.16	yes
9	FractionCSP3	0.3 (0.2)	-0.065	yes
10	BalabanJ	2.5 (0.5)	0.0003	no
11	Chi0	35.2 (14.8)	-0.18	no
12	Chi1	20.1 (7.7)	-0.18	no
13	Chi3v	3.6 (1.4)	-0.085	no
14	Chi4v	2.5 (1.2)	-0.071	no
15	HallKierAlpha	-2.2 (0.7)	-0.10	no
16	Kappa1	5.2 (1.8)	0.018	no
17	Kappa2	6.5 (2.7)	-0.058	no
18	Kappa3	3.2 (1.7)	0.022	no
19	BertzCT	1690.3 (703)	-0.20	no
20	NumAliphaticRings	1.6 (1.8)	-0.0032	yes
21	NumAromaticRings	2.0 (1.1)	-0.14	yes
22	NumAtoms	45.0 (18.0)	-0.18	no
23	MolWt	357.2 (116.8)	-0.13	no
24	MolMR	96.0 (30.0)	-0.17	no
25	MaxPartialCharge	0.3 (0.1)	-0.097	yes
26	MinPartialCharge	-0.4 (0.1)	0.22	yes
27	MaxAbsPartialCharge	0.4 (0.1)	-0.25	yes
28	NumChiralCenters	1.3 (2.2)	-0.17	yes

Table S6. Conformance of mean descriptor values to drug-like and lead-like filters.

#	Descriptor	Mean (std.dev.) value on the set	Ro5 threshold	Ghose threshold	Veber threshold	Ro3 threshold
1	MolLogP	3.3 (1.8)	5	within -0.4 .. +5.6		<=3
2	MolWt	357 (117)	500	within 180 .. 480		<300
3	NumHDonors	1.8 (1.3)	<=5			<=3
4	NumHAcceptors	4.3 (1.9)	<=10			<=3
5	NumRotatableBonds	5.4 (3.9)			<=10	<=3
6	MolMR	96 (30)		within 40 .. 130		
7	NumAtoms	45 (18)		within 20 .. 70		

Table S7. Coefficient values for the production SVM and LR models (9 descriptors).

#	Descriptors	SVM value (lower; upper bounds)[a]	LR value (lower; upper bounds)[a]
1	MolLogP	-0.25 (-0.41; -0.06)	-0.61 (-1.09; -0.16)
2	NumHAcceptors	0.08 (0.00; 0.29)	0.22 (0.00; 0.76)
3	NumHDonors	-0.09 (-0.25; 0.00)	-0.20 (-0.62; 0.00)
4	NumRotatableBonds	0.02 (-0.06; 0.21)	0.03 (-0.14; 0.49)
5	NumHeteroatoms	0.14 (0.00; 0.39)	0.32 (0.00; 0.94)
6	FractionCSP3	0.00	0.00
7	NumAliphaticRings	0.00	0.00
8	NumAromaticRings	-0.06 (-0.28; 0.00)	-0.07 (-0.65; 0.00)
9	MaxPartialCharge	-0.09 (-0.25; 0.00)	-0.19 (-0.59; 0.00)
10	MinPartialCharge	0.00	0.00
11	MaxAbsPartialCharge	-0.12 (-0.28; 0.00)	-0.31 (-0.68; 0.00)
12	NumChiralCenters	-0.11 (-0.33; 0.00)	-0.19 (-0.77; 0.00

[a] Confidence intervals at 95% level obtained using the bootstrap method.

Table S8. Predictions made by classification SVM and LR models for the prospective molecule list.

#	Structure	SVM, activity	SVM, activity rank*	SVM, predicted value	LR, activity	LR, activity rank*	LR, predicted value
1	KUD1008	0	18	-0.03	1	17	0.19
2	KUD1022	0	19	-0.05	0	20	-0.13
3	KUD1036	1	16	0.14	1	15	0.35
4	KUD1044	1	8	0.25	1	8	0.61
5	KUD1050	0	24	-0.50	0	24	-1.08
6	KUD1066	1	6	0.30	1	6	0.69
7	KUD1130	1	5	0.36	1	7	0.68
8	KUD1132	1	14	0.21	1	13	0.50
9	KUD1133	0	22	-0.12	0	22	-0.31
10	KUD1134	1	12	0.23	1	14	0.47
11	KUD1135	0	20	-0.07	0	21	-0.18
12	KUD138	1	2	0.52	1	2	1.21
13	KUD165	1	9	0.24	1	10	0.54
14	KUD224	1	11	0.23	1	12	0.53
15	KUD225	1	10	0.24	1	11	0.54
16	KUD233	1	17	0.06	1	18	0.18
17	KUD529	1	15	0.15	1	16	0.33
18	KUD530	1	13	0.23	1	9	0.56
19	KUD649	1	3	0.44	1	4	1.07
20	KUD718	1	1	0.69	1	1	1.68
21	KUD759	0	23	-0.16	0	23	-0.33
22	KUD833	1	4	0.43	1	3	1.17
23	KUD834	1	7	0.27	1	5	0.75
24	KUD990	0	21	-0.08	0	19	-0.06

* 1 – the most active structure, 24 – the least active structure according to prediction.

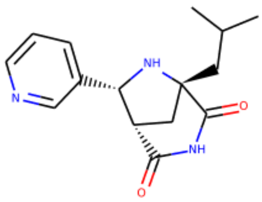
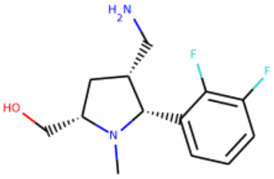
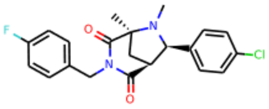
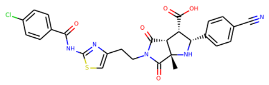

KUD718 KUD138 KUD649 KUD833

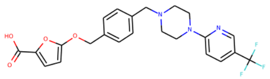
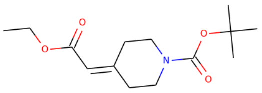
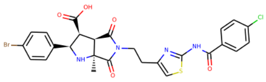
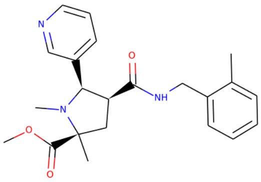

KUD1066 KUD1130 KUD834 KUD1044

Figure S6. The most active predicted by SVM and LR models molecules. Each structure is represented with a random enantiomer, indistinguishable by the classification models used.

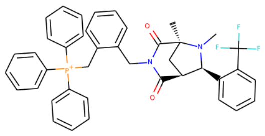
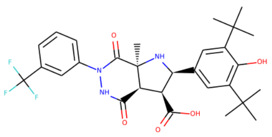
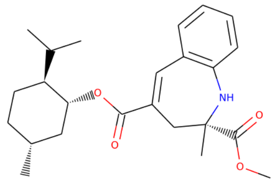
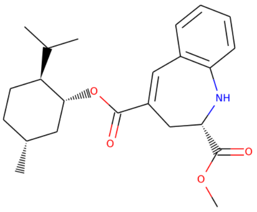

KUD1050 KUD759 KUD1133 KUD1135

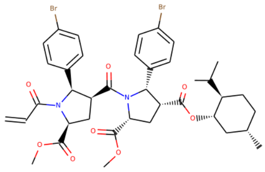
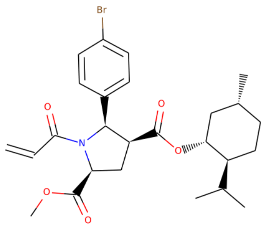
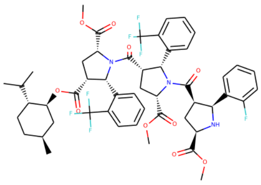

KUD990 KUD1022 KUD1008*

Figure S7. The most probably inactive molecules predicted by SVM and LR models. Each structure is represented with a random enantiomer, indistinguishable by the classification models used.
*KUD1008 was predicted active according to LR model.

Table S9. Predictions made by regression LASSO and SVR models for the prospective molecule list.

#	Structure	Lasso, log (Atc., M)	Lasso, LE	SVR, log (Act., M)	SVR, LE
1	KUD1008	0.6	-0.01	-3.4	0.07
2	KUD1022	-1.8	0.07	-2.6	0.11
3	KUD1036	-2.6	0.11	-3.2	0.14
4	KUD1044	-2.7	0.13	-2.7	0.13
5	KUD1050	-2.0	0.06	-3.2	0.09
6	KUD1066	-3.4	0.14	-3.2	0.13
7	KUD1130	-4.1	0.30	-3.6	0.26
8	KUD1132	-3.0	0.17	-4.5	0.26
9	KUD1133	-2.9	0.14	-3.1	0.15
10	KUD1134	-3.7	0.22	-4.2	0.25
11	KUD1135	-3.0	0.15	-3.3	0.16
12	KUD138	-3.3	0.26	-4.6	0.36
13	KUD165	-3.0	0.21	-3.9	0.27
14	KUD224	-3.2	0.22	-3.6	0.25
15	KUD225	-3.0	0.21	-3.8	0.26
16	KUD233	-2.9	0.18	-3.3	0.20
17	KUD529	-2.5	0.10	-3.4	0.14
18	KUD530	-2.7	0.13	-3.6	0.17
19	KUD649	-2.6	0.13	-3.9	0.20
20	KUD718	-3.5	0.24	-3.6	0.25
21	KUD759	-2.7	0.09	-3.3	0.11
22	KUD833	-2.7	0.10	-3.0	0.11
23	KUD834	-2.3	0.08	-3.0	0.11
24	KUD990	-0.2	0.01	-3.4	0.09

* For activity coloring log(Act., M) value of -3.0 is taken for white; for LE coloring 50% percentile for both column values is taken for white. $LE=-0.6 \cdot \ln(10) \cdot \log(Act., M)/NH$ [$kcal \cdot mol^{-1} \cdot atom^{-1}$], where NH – is the number of heavy atoms. That conforms to the generally accepted definition of $LE=-\Delta G/NH=-RT \cdot \ln(Act)/NH$.

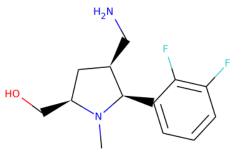

KUD138

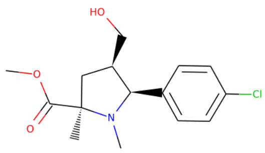

KUD165

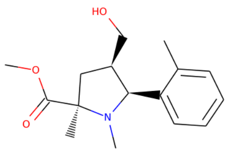

KUD224

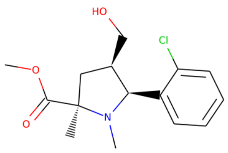

KUD225

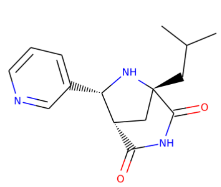

KUD718

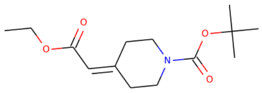

KUD1130

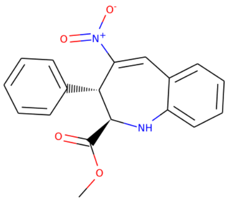

KUD1132

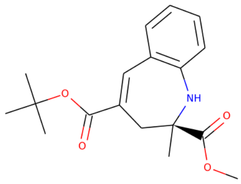

KUD1134

Figure S8. The most active and most efficient (high LE) molecules according to consensus LASSO and SVR models predictions. Each structure is represented with a random enantiomer, indistinguishable by the regression models used.

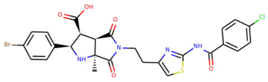

KUD834

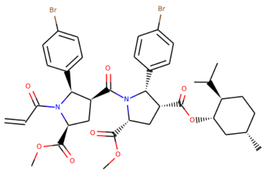

KUD990

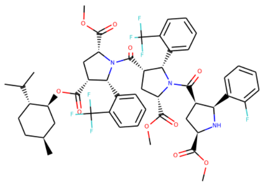

KUD1008

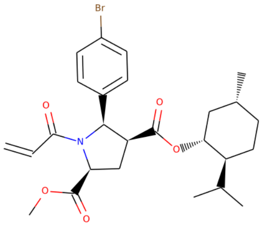

KUD1022

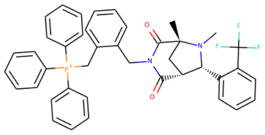

KUD1050

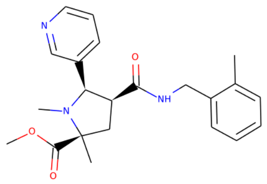

KUD1044*

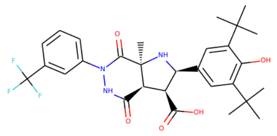

KUD759**

Figure S9. The least active and least efficient (low LE) molecules according to consensus LASSO and SVR models predictions. Each structure is represented with a random enantiomer, indistinguishable by the classification models used. * present in the least active molecules. ** present in the least efficient molecules.

Table S10. The favorable binding modes according to AutoDock Vina docking for Ro3 subset of active ChEMBL molecules. SrtA model – PDB:2KID. Potential hydrogen bond (electrostatic) interactions of the ligands with Arg197 are highlighted, the distances are between heavy atoms in Å.

Num	ChEMBL id	Surface	Bonds
1	CHEMBL1288785	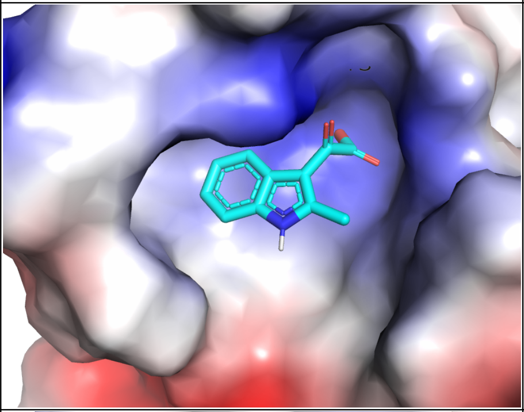	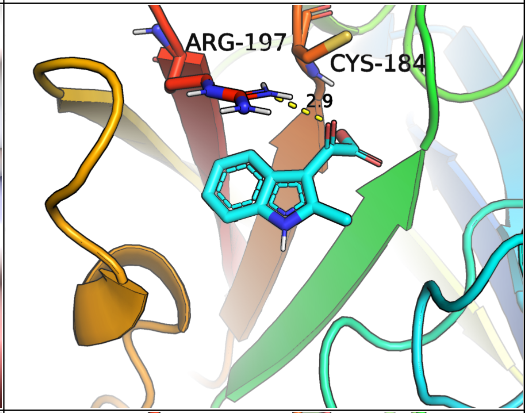
2	CHEMBL165058	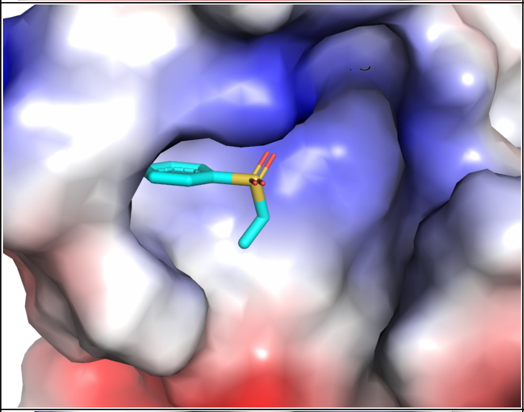	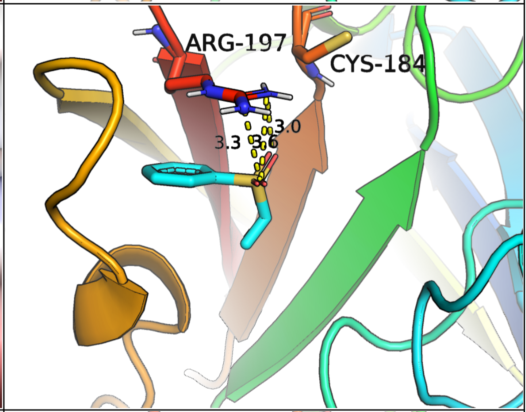
3	CHEMBL234180	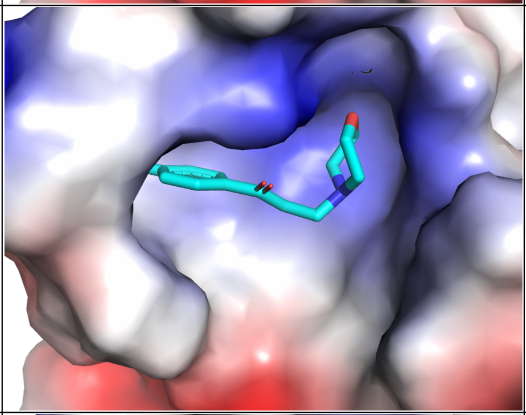	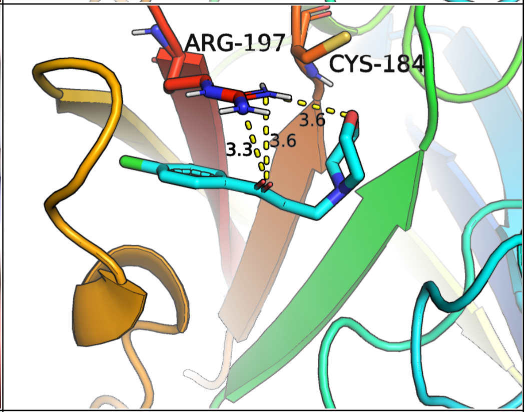
4	CHEMBL258985	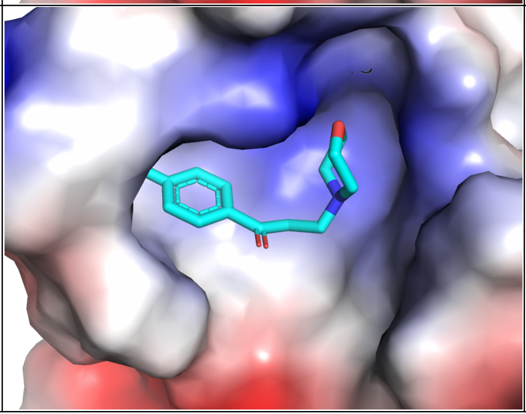	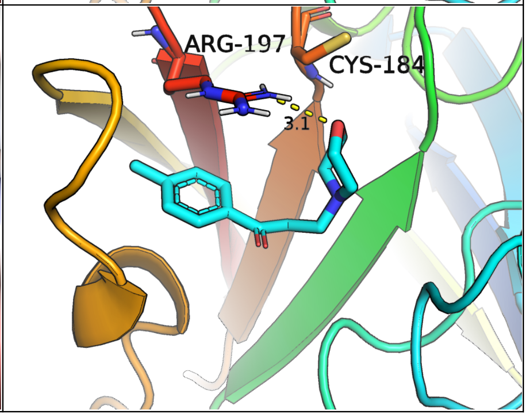

5	CHEMBL260814	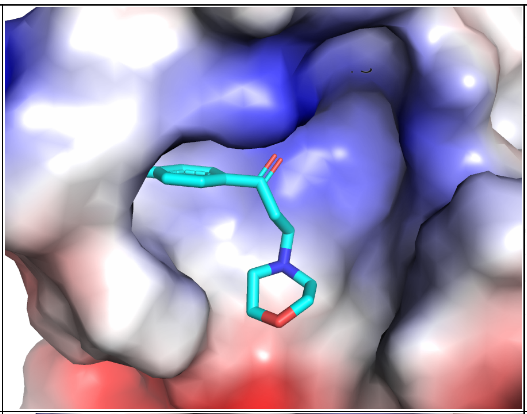	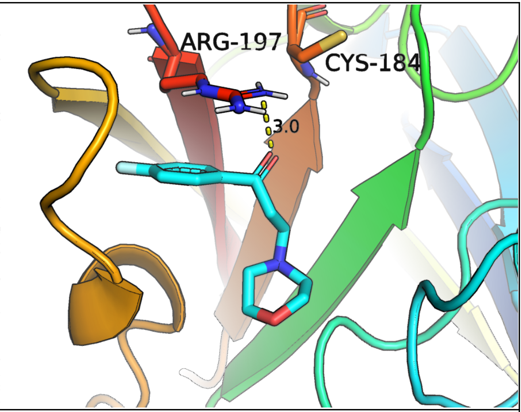
6	CHEMBL356828	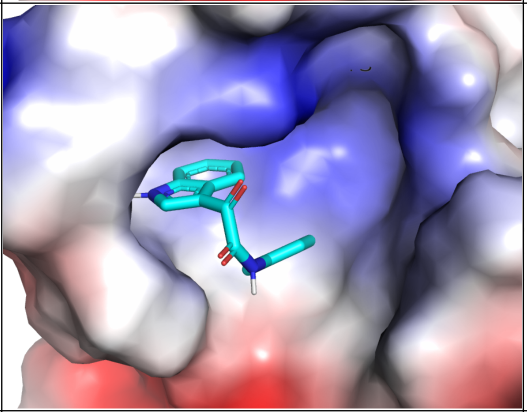	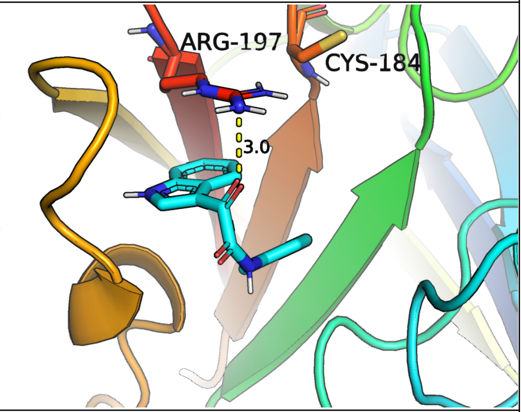
7	CHEMBL405826	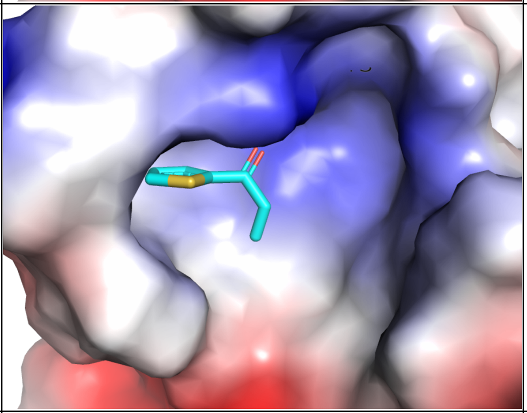	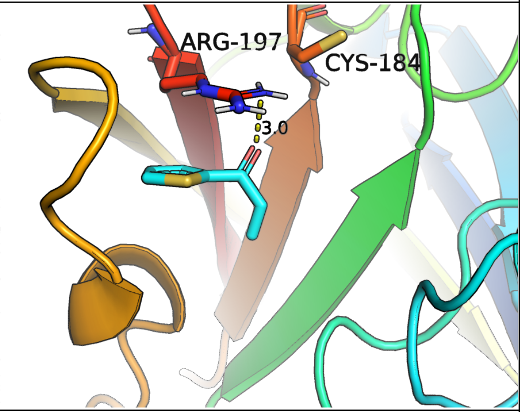
8	CHEMBL407492	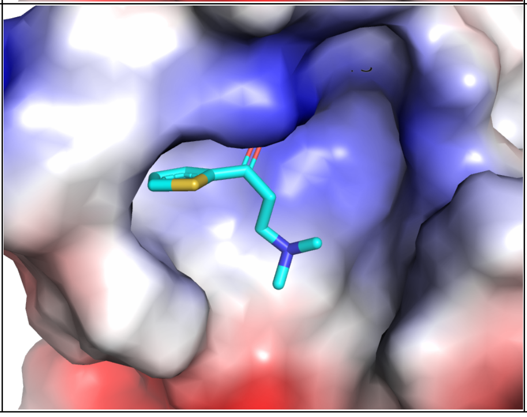	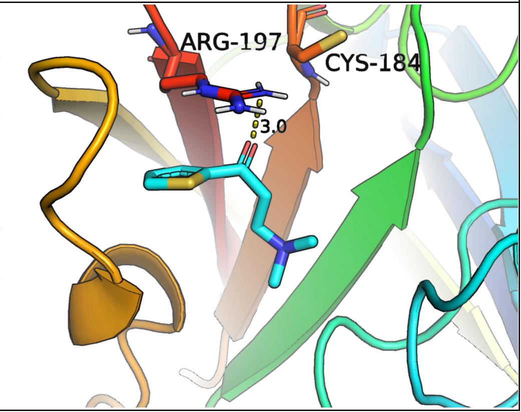

9	CHEMBL57285	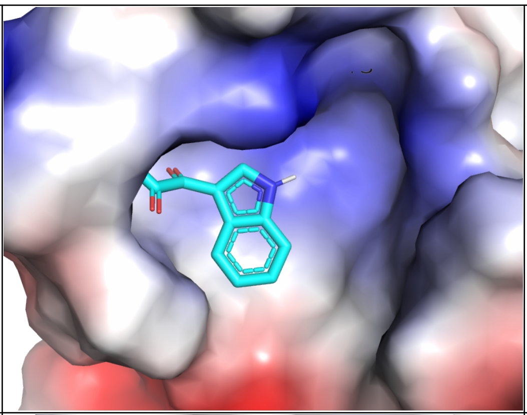	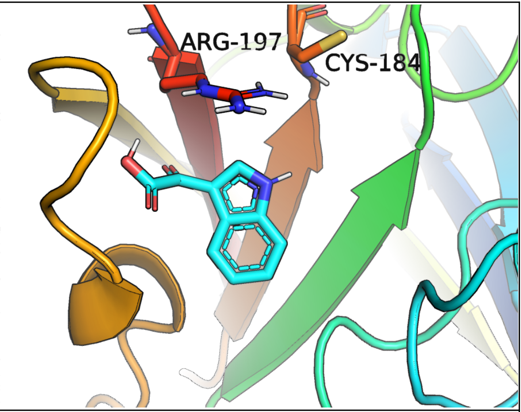
10	CHEMBL78775	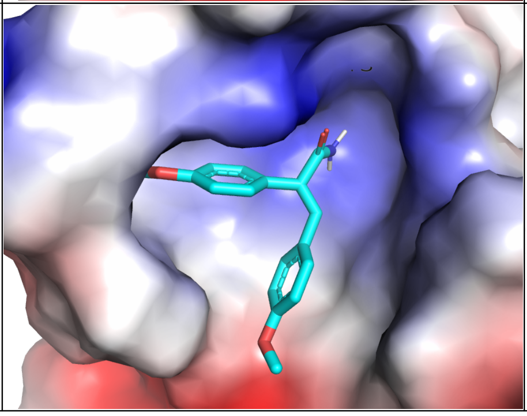	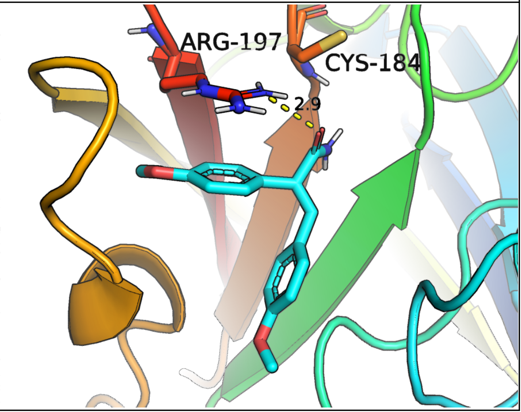
